# Working With Environmental Noise and Noise-Cancelation: A Workload Assessment With EEG and Subjective Measures

**DOI:** 10.3389/fnins.2021.771533

**Published:** 2021-11-01

**Authors:** Kerstin Pieper, Robert P. Spang, Pablo Prietz, Sebastian Möller, Erkki Paajanen, Markus Vaalgamaa, Jan-Niklas Voigt-Antons

**Affiliations:** ^1^Quality and Usability Lab, Institute of Software Engineering and Theoretical Computer Science, Electrical Engineering and Computer Science, Berlin Institute of Technology, Berlin, Germany; ^2^German Research Center for Artificial Intelligence, Berlin, Germany; ^3^Tampere Wireless Headset Audio Lab, Finland Research Center, Huawei Technologies Oy (Finland) Co., Ltd., Tampere, Finland

**Keywords:** mental workload, ambient noise, noise-canceling, event-related potentials, EEG frequency, subjective measures

## Abstract

As working and learning environments become open and flexible, people are also potentially surrounded by ambient noise, which causes an increase in mental workload. The present study uses electroencephalogram (EEG) and subjective measures to investigate if noise-canceling technologies can fade out external distractions and free up mental resources. Therefore, participants had to solve spoken arithmetic tasks that were read out via headphones in three sound environments: a quiet environment (*no noise*), a noisy environment (*noise*), and a noisy environment but with active noise-canceling headphones (*noise-canceling*). Our results of brain activity partially confirm an assumed lower mental load in *no noise* and *noise-canceling* compared to *noise* test condition. The mean P300 activation at Cz resulted in a significant differentiation between the *no noise* and the other two test conditions. Subjective data indicate an improved situation for the participants when using the noise-canceling technology compared to “normal” headphones but shows no significant discrimination. The present results provide a foundation for further investigations into the relationship between noise-canceling technology and mental workload. Additionally, we give recommendations for an adaptation of the test design for future studies.

## 1. Introduction

In flexible working surroundings like landscape offices, business trips, or even the home office, people have to deal with noisy environments. It is hardly avoidable to be distracted by, e.g., other conversations, traffic noise, or screaming kids while focusing on the actual task. The combination of stressful influences and task difficulty increases the workload for the person. The interaction of task characteristics and the person's capacity influences the amount of mental load a person is able to allocate in a task (Choi et al., 2014). Additionally, environmental stressors decrease task performance and lead to motivational deficits (Evans and Stecker, 2004). In task solving, which requires cognitive resources, keeping the demand on an appropriate level is important. Especially while working, a balance is necessary between the work and any parallel (and potentially distracting) tasks to stay focused over longer time (Teigen, 1994). There is evidence for a relationship between the development of mental disorders and continuous high levels of workload, as well as for decreased satisfaction and well-being (van Daalen et al., 2009).

Mobile solutions which help to stay focused are frequently used to improve the situation for the working person. One option to directly reduce environmental auditory noise without changing the working environment is headphones with active noise-canceling. These technologies use a basic principle of wave optics called destructive interference. A signal superimposes the incoming noise signal, which has the same amplitude but the opposite phase (Kuo et al., 2006). Thereby, noise-canceling headphones offer an individual and a mobile solution for noise suppression. In the present study, we examine to what extent noise affects the workload level in task solving and whether noise-canceling technologies can reduce the workload compared to the use of headphones in normal mode in otherwise identical circumstances.

In the present experiment, the participants performed the same cognitive task in three different noise environments, which serve as test conditions. The within-subject test design should deliver insights about differences in the workload level between conditions and changes over time for each condition separately. In the *no noise* condition, the quiet environment should allow the participants to focus on the task. We suggest the mental load to be on a mid-level in this test condition. The ambient noise presentation was assumed to increase mental load due to a higher need for resources to stay focused. This effect should become sharper in the *noise* condition as the persons were directly exposed to the ambient noise. With the activation of the noise-canceling feature in the *noise-canceling* condition, the workload level was supposed to be lower compared to the *noise* condition and slightly increased compared to *no noise* condition.

For measuring workload, we employed subjective and EEG measurement of brain. Subjective measures primarily assess the participants' reactions to experimental manipulation and thereby give valuable insights about the person's state at the moment of the measurement. EEG and, in general, physiological measures offer the advantage of a recording over an experiment's whole duration. The resulting continuous signal enables the detection of stimulus-related reactions and also the observation of changes over time. Therefore, the combination of measures is assumed to give more complex insights as one measure alone. We suggested the delivered findings from subjective measures to give a fundamental differentiation between conditions into the person's mental and affective state. EEG should deliver information about the brain's underlying processes, which cause differentiation in the level of mental workload for the three test conditions.

Research about workload and its underlying processes was extensively studied for decades, but it is still an elusive concept. There are different considerations about how to define workload and how it interacts with other mental processes. In an early concept, given by Kahnemann, “mental effort” is described as a capacity that is invested in task processing or demanded by a task. The extend of effort invested in task solving is less influenced by the task solver's intention, but rather it is regularized by the task demand (Kahneman, 1973). Later on, Wickens describes “Workload” as the interrelation between the task demand and the humans' limited mental resources needed for solving it. Depending on the complexity of one or more tasks, multiple resources are required. These resources are multidimensional and can be differentiated in several “stages” and “modalities” (Wickens, 1979, 2008), whereas the resulting load is a global (mental-) “load” on the human (Rasmussen, 1979; Wickens, 2008). Especially subjective measures have a high operator acceptance because of paying attention to the opinion of the participant (Hill et al., 1992). Since it appears that emotions are related to the perceived workload of a task and the other way around (Jeon et al., 2011; Chaouachi and Frasson, 2012) we wanted to investigate aspects of the emotional state of the participants in the current test design. With higher ratings for negatively related emotional items, we suggest a higher perceived workload.

In several studies, it was shown that both subjective measurements and EEG measurements show sensitivity for workload (Parasuraman, 1990; Hankins and Wilson, 1998; Borghini et al., 2014). On the one hand, it is of interest to confirm the results of one measurement with the other measurement results. However, it is also suggested that subjective meaning conscious ratings deliver deviating observations as unconscious activation in the brain. We expect additional and possibly more detailed observations from the study of brain activity.

EEG data can be investigated regarding mental processing and workload, considering event-related potentials (ERPs) and power spectral densities of frequency bands. In the frequency domain, we investigate the spectral power of frequency bands. The EEG frequency bands of interest are delta, theta, alpha, beta, and gamma. We define the frequency range 0.1–4 Hz corresponding to delta (delta is categorized differently but often in the range between 0.3 and 4.5 Hz; see, e.g., Feinberg et al., 1987; Anderson and Horne, 2003; Knyazev, 2012), 4–8 Hz corresponding to theta, 8–12 Hz corresponding to alpha, 12–30 Hz corresponding to beta, and 30–40 Hz corresponding to gamma (gamma frequency range is referred to as < 30*Hz*; see, e.g., Knyazev, 2012). An increase in delta activation was observed in pilots during flying operations with rising cognitive demand (Harmony et al., 1996; Wilson, 2002). The theta band is suggested to be associated with memory processes and mental workload (Klimesch, 1999). Reduced alpha in combination with higher theta power is suggested to occur when workload increases (Brouwer et al., 2012). With increasing task difficulty and thereby with increasing cognitive load, the frontal-midline theta responds with a maximum at frontal central electrode positions (Ishihara and Yoshii, 1972; Gevins et al., 1998). The alpha band is the dominant frequency in the human scalp EEG (Klimesch, 1999). Alpha band power response to workload showed a varying behavior. In a visual spatial task, alpha at parietal-temporal-occipital region decreased with task difficulty (Gevins et al., 1998). In a following experiment in which a memory component extended the task, it was shown that alpha total power increased with task difficulty (Murata, 2005). In an experiment by (Yu et al., 2009) with a mental arithmetic task, an alpha decrease and a beta increase at parietal and occipital sites was shown. Additionally, beta power activation seems to be related with cognitive processing (Ray and Cole, 1985). Studies investigating the gamma band suggest an increased activation with raised task difficulty (Gevins et al., 1998; Knoll et al., 2011). Other findings suggest gamma (40 Hz) activity reported an activation in a selective attention task of auditory stimuli at the auditory cortex (Tiitinen et al., 1993). It is also known to be more generally associated with sensory processing and cognitive processes with a wide distribution on the scalp (Başar-Eroglu et al., 1996).

Based on these insights, we suggested the highest delta, theta, beta, and gamma power spectral density in the *noise* condition and in the *no noise* condition the lowest. In the *noise-canceling* condition, it was suggested to be on a mid-level. For alpha power spectral density, it was suggested to be in lower in the *no noise*, on a mid-level in the *noise-canceling*, and smallest in the *noise* condition.

In the time domain, the stimulus-locked ERPs were investigated. Based on a body of literature, we suggested differences in the P300 that is a positive component of the ERPs, which peaks 300 ms after a stimulus onset (Duncan et al., 2009). It shows sensitivity to workload, a maximum characteristic over midline scalp sites, and has a centro-parietal distribution. The P300 component is often divided in two parts: the P3a and the P3b. Whereby, the P3a appears as a response to novelty of a stimulus and as an orienting response, and the P3b shows a sensitivity for task-relevant processing and decision-making processes (Friedman et al., 2001). The amplitude of P300 has been reported to be an indicator of different levels of difficulty (Wickens et al., 1977; Kramer et al., 1987) and thereby workload (Ullsperger et al., 2001). With increasing workload, the amplitude of P300 is suggested to remain smaller, whereby the latency of the component remains higher (Duncan et al., 2009). Studies of the P300 were conducted primarily in conjunction with a classical Oddball paradigm. With our task design, we deviate from the classic oddball like it was done in studies about the discrimination of different workload levels (Ullsperger et al., 2001; Allison and Polich, 2008). The main task, solving mental arithmetics, is complicated with different levels of noise intensities. This scenario is comparable to attending an online meeting in a noisy environment while recording thoughts into a protocol. The mental demand caused by the recall of numbers and the calculation of the arithmetics is suggested to elicit a P300. The spoken arithmetic equations and the environmental noise address the same sensory modality, which means a higher workload in this channel. So we suggest the P300 amplitude to be smallest in the *noise* and most extensive in the *no noise* condition. As the *noise-canceling* technology suppresses the ambient noise, making it easier to focus as in the *noise* condition, we suggested the amplitude to be on a mid-level (between the other two conditions).

In the following, we explain the task and the whole test setup. The following part reports the results and delivers the base for the subsequent discussion, including limitations and suggestions for future work. In the final section, we provide a conclusion.

## 2. Materials and Methods

### 2.1. Task

The fundamental task of each trial was to solve an arithmetic equation. These consisted of two numbers and the four basic operators: addition, subtraction, multiplication, and division. The two numbers and the result were in the range of 1–200, and they were all integer numbers. These tasks were presented auditory via headphones to the participant.

We decided against providing the participants with a fixed time for answering because the difficulty among the tasks varied heavily. In pre-tests, we observed participants to develop answering strategies to cope better with the demanding situation. For example, the task 3 + 2 was more intuitive to solve in a short time for most participants, whereas many people took a long time to solve 23 * 7. While testing a constant time given for all sorts of tasks, we noticed that participants sometimes typed in the answer of an easy task but waited until the time was almost over to provide themselves with a short break. If we are now interested in, e.g., the total amount of tasks solved correctly throughout a condition, this avoiding behavior will bias our findings. Furthermore, the short, unplanned breaks might impact the perceived workload as well. Hence, we decided to create a machine learning-based algorithm to predict the ideal time needed to solve the task for each participant. Since it is not the focus of this paper to describe the algorithms in-depth, we present here only the fundamental idea of the model: Using general features from the arithmetic task at hand, e.g., the operator and the digit-span, as well as the previous performance of the participant, we predicted the time per task individually. This allowed us to address the individual abilities of each subject but also to not allow for any headroom in the time given.

### 2.2. Subjective Measures

We chose the NASA Task Load Index (NASA-TLX) questionnaire to assess the participants' perceived workload. It is a sensitive indicator of workload because participants describe their personal impressions from their individual viewpoint (Hart and Staveland, 1988). It is a widely acknowledged multi-dimensional rating scale, which was adapted in several studies (Hart, 2006) to obtain workload estimates. In the present study, ratings from six dimensions (mental demand, physical demand, temporal demand, frustration, effort, and performance) were averaged without individual weights. We decided on the unweighted version as it is easier to apply, and the sensitivity seems to be similar as with adding the weighting process (Hart, 2006). Since our approach was to get information about the participants' affective states, we used the Self-Assessment Manikin (SAM). The pictorial assessment is easy to explain and covers essential aspects of a person's affective reaction to a stimulus (Bradley and Lang, 1994). The participant could rate with three items: pleasure (from 1 = satisfied to 9 = unsatisfied), arousal (from 1 = excited to 9 = unexcited), and dominance (from 1 = controlled to 9 = controlling). Additionally, a scale for assessing subjectively experienced effort was deployed. We referred to this scale as “subjective rating scale (SRS).” The scale is an adaption from the SEA scale (Eilers et al., 1986) and measures the subjectively experienced effort for performing the task. We transferred the original scale into a numeric rating scale with equal intervals starting from 1 (“little effort”) to 7 (“extreme effort”).

### 2.3. EEG

EEG data were continuously recorded from 14 standard scalp locations according to the 10–20 system (Oz, O1, O2, P3, P4, Pz, Cz, C3, C4, Fz, F3, F4, T3, T4). Since high-density EEG measurements are often time consuming and unpleasant for the test person due to the high number of electrodes, we aimed for a reduced test setup that still delivers informative value. Kumar and Kumar (2016) measured cognitive load by using EEG and found reliable results with 14 channels (similar done by Anderson et al., 2011). An even reduced number of channels was used in studies by Brouwer et al. (2012) and Hogervorst et al. (2014), in which they investigated workload with not more than seven channels successfully. Given that the expected effects get visible at different regions throughout the whole scalp characteristics and considering potential noisy channels, we decided against a minimum but a reduced setup of 14 channels.

### 2.4. Test Setup

The hearing ability of each participant was tested to ensure a comparable experience of the auditory stimuli for every participant. This was done using an audiometry tool (model MA 33; MAICO Diagnostics GmbH, Berlin, Germany). Baseline instructions and both tasks were deployed in PsychoPy (Peirce et al., 2019) running on a ThinkPad X1 Carbon Ultrabook (Lenovo Ltd., Hongkong). All visual stimuli were presented on a Fujitsu (model: DY24W-7) monitor. The acoustic representation of the mathematical equations was generated by the Win TTS API (German language) and provided to the participants via Sony WHX-1000X M3 headphones in 70 dB SPL. In two out of the three condition blocks, the noise was presented to the participants via four loudspeakers (model PM 0.4) from Fostex (Foster Electric Co., Ltd., Tokyo, Japan) with 76 dB SPL. They were mounted on stands at a height of 1.0 m, placed at a 1.5 m distance to the participants and at a 90° angle to each other. The audio file of the background noise was controlled from a notebook (model Vaio VPCF13C5E; Sony Corporation, Tokyo, Japan) with the expansion card (model HDSP I/O ExpressCard; RME Intelligent Audio Solutions, Audio AG, Haimhausen, Germany). Noise consisted of a combination of very frequent numbers (using the same TTS voice as for the tasks; partially overlapping from different directions), environmental noise (recordings from cars, public streets, cafe chatter; from every direction, not overlapping), and speech snippets (excerpts of German podcasts and news broadcasts; partially overlapping from different directions).

EEG data, stimulus marker (e.g., keypresses of the participant), and stimulus data from PsychoPy was time-synchronized and recorded as one combined data stream via Labstreaminglayer Framework in Lab Recorder running on a ThinkPad X1 Carbon Ultrabook (Lenovo Ltd., Hongkong, China). To access EEG data in Labstreaminglayer, we used g.USBamp App^1^ (Pre-release 30.04.2019). For streaming stimulus marker from PsychoPy, we used pylsl^2^ (version 1.13.1).

EEG was assessed via wet Ag/AgCl electrodes placed in a head cap, a driver box for 16 channels and the g.USBamp amplifier by g.tec (g.tec medical engineering GmbH, Schiedlberg, Austria).

**Figure 1:**
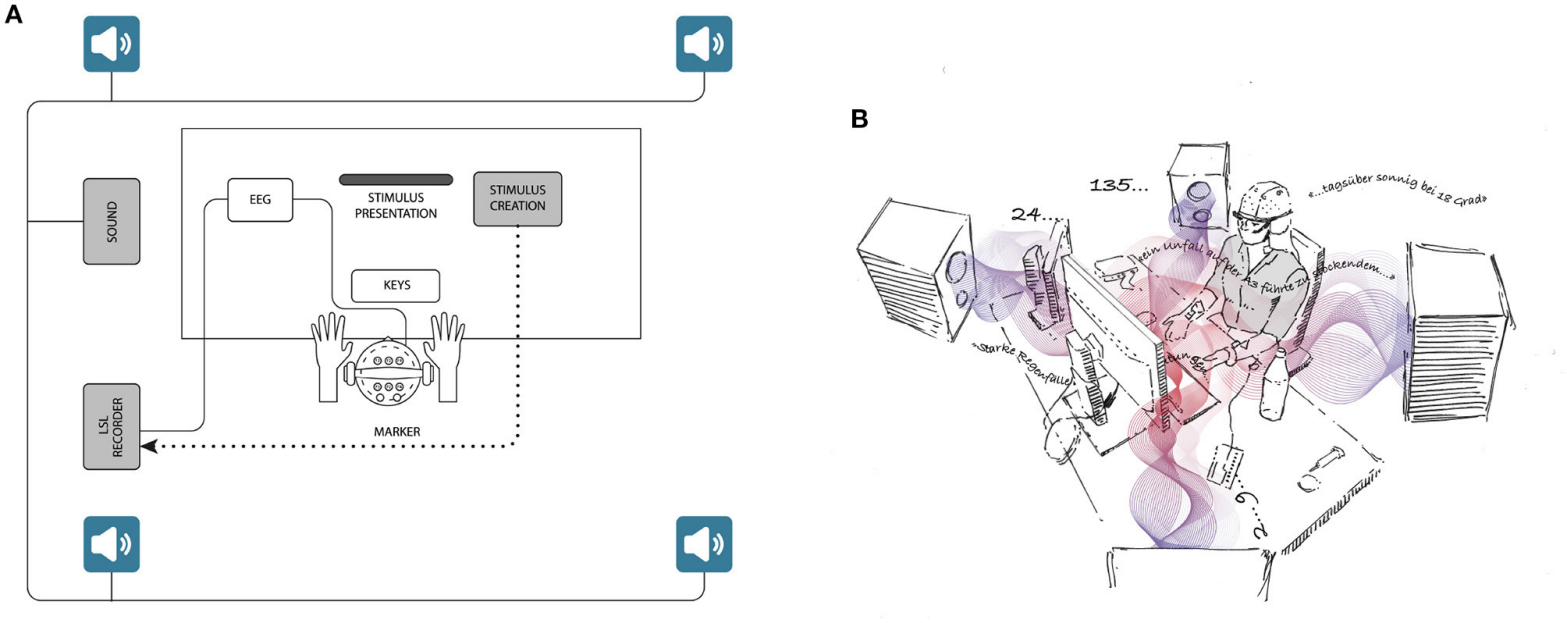
**(A)** Schematic presentation of the whole test setup, including measurements, stimulus presentation, and the acoustical setup. Four loudspeakers were mounted on stands at the height of 1.0 m, placed at a 1.5 m distance to the participant and in at a 90° angle to each other. **(B)** Illustration of the environmental noise situation in the *noise* and *noise-canceling* condition. It consisted of a combination of frequent numbers, environmental noise, and speech snippets.

EEG data and stimulus marker data (e.g., keypresses of the participant) from PsychoPy were time-synchronized and recorded as one combined data stream via Labstreaminglayer Framework in Lab Recorder running on a notebook ThinkPad X1 Carbon Ultrabook (Lenovo Ltd., Hongkong). Figure 1A shows the apparatus with all measures, the stimulus presentation, and arrangement of loudspeakers. Also the environmental noise in the setup is illustrated in Figure 1B. For analyzing the EEG data, we used the open-source Python package MNE (Gramfort et al., 2013) (version 0.19.1). Statistical analysis was computed with the open-source package Pingouin (Vallat, 2018) (version 0.3.7).

### 2.5. Procedure

**Figure 2:**
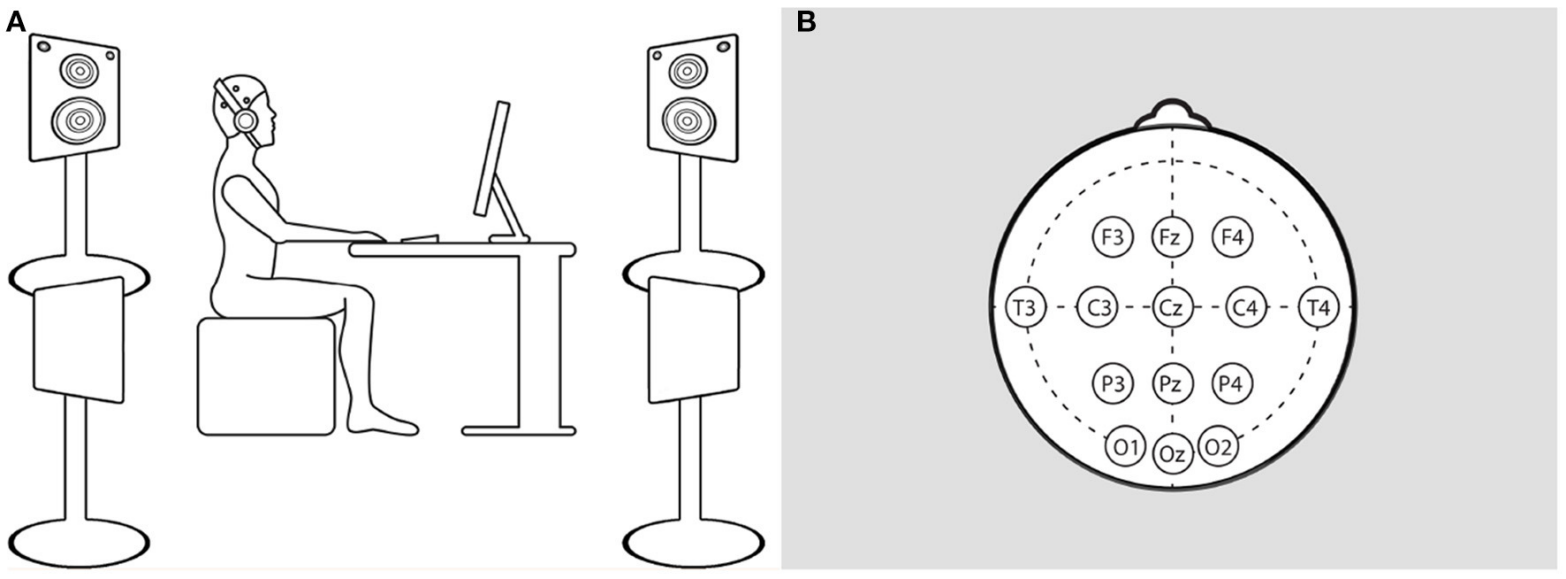
Schematic presentation of the participants' seating position **(A)** and the electrode setup of EEG cap **(B)**. It should be noted that on the left illustration **(A)**, the height of the loudspeakers was adjusted for display purposes but, as mentioned, was actually 1.0 m from the floor. **(B)** The distribution of applied electrodes on the scalp.

The experiment was conducted in a quiet standardized test room adhering to ITU-T Rec. P.910^3^ and P.911^4^. The participants were seated in a chair with a comfortable and upright seating position for the whole duration of the test. In preparation for the EEG measurement, the experimenter placed a flexible cap with plugged-in electrodes on the participant's head and inserted a water-based conductive gel in every electrode. The preparation was completed by equipping the participants with headphones. Figure 2 illustrates the participants' seating position and the arrangement of applied electrodes on the scalp. To compare individual responses to different sound environments, the participants had to perform the task in three different conditions: *no noise* (quiet environment), *noise* (noise environment), and *noise-canceling* (noise environment with the noise-canceling function of headphones). The order of the three condition blocks was randomized. Additionally, the order of the condition was counterbalanced [6 possible combinations; count of every combination (*M* = 4.67, *SD*:1.03)]. Each block followed the same procedure: First, the participant had to perform the three subjective measure ratings: NASA-TLX, SAM, and the subjective rating scale. Each questionnaire was presented in a separate view on display in front of them. The ratings were submitted by moving a slider for each item. After the subjective measures, the main task block started and had a duration of 30 min. After the main task, the participants were again asked to rate their state with the subjective measure questionnaires. Between the condition blocks, the participants should rest for 5 min.

### 2.6. Participants

In total, 29 persons aged 21–64 years (*M* = 34.62, *SD* = 12.62) participated. The gender distribution was nearly balanced (male: 15, female: 14). All participants stated that they were employed, studying at university, or both at that moment. Participants were recruited via a university participant database. All of them confirmed that they had a normal or corrected to normal vision and average hearing ability. None of the participants showed a hearing impairment in the performed audiogram (see section 2.5). For participation, the persons got monetary compensation; 15 euro per hour and a bonus depending on their performance in solving the arithmetic tasks (beginning with 50% performance score: 5 euro to max. 10 euro in case of about 100%). This was done to try to get the participants more motivated to gain correct results. The participants were informed about the experiment beforehand, and they agreed to it by signing the informed consent sheet. The study abides by the standards specified in the Declaration of Helsinki. The Ethics Committee of the Faculty IV of Technical University Berlin evaluated the procedure retrospectively and declared that all ethical aspects of the study design follow the Guideline of the German Research Foundation (date of assessment: 17.02.2021; fast track code: FR_2021_01retro).

### 2.7. Data Analysis

#### 2.7.1. Subjective Measures

The ratings for each item of the subjective measures were collected before and after the task in each test condition block. Values from the beginning of the test condition block were subtracted from ratings after to gain baseline corrected values. The differences between conditions were of interest. By normalizing the values, we measured only changes in ratings induced by the task and the noise situation. All “before” ratings were performed in an equal quiet surrounding. Although the test condition block order was randomized for every participant to avoid sequence effects, we could not exclude that specific block sequences might affect the dependent variable. Therefore, we considered the block order as within factor. Consequential, a two-way repeated measurements ANOVA with the within factors “condition” and “block order” was computed for each questionnaire item (Subjective rating scale: 1 item, SAM: 3 items, and NASA-TLX: 6 items). Significant main effects are reported with a Greenhouse-Geisser corrected *p*-value. The *post-hoc* comparisons were corrected with a Bonferroni–Holm adjustment.

#### 2.7.2. EEG Acquisition and Processing

EEG was continuously recorded from 16 channels (for details, see section 2.5) and sampled with 265*Hz*. The impedance between EEG electrodes and the scalp was kept under 5 kω. One data set had to be excluded from analysis due to an incorrect time synchronization of the stimulus marker stream coming from Psychopy and the measured EEG data stream. In total, data from 28 subjects were included in the EEG analysis. The raw data were filtered with a fir (Filter design: Firwin) band-pass filter from 0.1 to 45*Hz* with a Hamming window. The filter length was 8, 449 samples (33.004*s*). Additionally, a fir (Filter design: Firwin) band stop filter with a Hamming window with 50*Hz* was applied. The aim was to exclude high-frequency line-noise coming from electrical equipment and to remove slow drifts. Channels with extreme noise were detected by visual inspection and removed from the data set and interpolate afterward. Interpolation of bad channels in MNE is done with the spherical spline method (Perrin et al., 1989) that computes the missing signal based on the location and the data of the remaining channels. Afterward, the filtered data were re-referenced to an average reference.

#### 2.7.3. Artifact Rejection With SSP

For removing noise coming from eye movements (EOG) and heart activity (ECG), we chose Signal-Space Projection (SSP) (Uusitalo and Ilmoniemi, 1997). Therefore, we defined one channel that showed the corresponding artifacts' most characteristic behavior. The computation of the SSP projectors was done on the filtered and re-referenced continuous data of one participant and per condition separately. For the calculation of the EOG projector, the data were band-pass filtered from 1 to 10 Hz (filter design: Firwin; Window: Hann window) to remove DC offset and distinguish blinks from saccades. On the basis of blink detection creating events, the SSP projectors were computed. After that, the data were filtered in one contiguous segment from 1 to 35 Hz [filter design: Firwin; Window: Hamming window; Filter length: 2, 560 samples (10.000*s*)]. For creating the ECG projector, data were band-pass filtered from 5 to 35 Hz [filter design: Firwin; Window: Hann window; Filter length: 2,560 samples (10.000*s*)]. After the computation of the ECG projector, the data were filtered with a band-pass filter [filter design: Firwin; Window: Hann window; Filter length: 2, 560 samples (10.000*s*)] in one contiguous segment from 1 to 35 Hz. The computed EOG and ECG SSP projectors were saved and applied in further processing steps.

#### 2.7.4. Time Frequency Analysis

For analysis in the frequency domain, we segmented the continuous data into epochs. The beginning audio output of the math equation served as stimulus onset. Depending on the number of solved tasks a participant reached per block, the number of trials varied. All these events were used to create epochs. Every trial epoch started 200*ms* before the event and ended 3800*ms* after the stimulus onset. The 3800*ms* after stimulus onset corresponds to the maximum audio output duration of the longest equation. On the epoched data, we applied the formerly calculated SSP projectors to remove ECG and EOG artifacts. We then calculated the periodograms from 0.1 to 45*Hz* using Welch's method (Welch, 1967) with a sliding hamming window and a window size of 1.0*s* (256 samples), which were then averaged for each channel and epoch. The calculation was done for every participant and each condition block separately. The resulting power spectral densities (PSDs) were normalized by dividing each power value by the total power (per condition block). The correction was done for each participant individually to consider inter-individual variations. Afterward, we aggregated the PSDs of the corresponding EEG frequency bands: delta: 0.1–4 Hz, theta: 4–8 Hz, alpha: 8–12 Hz, beta: 12–30 Hz, and gamma: 30–45 Hz. To compare for differences between conditions, we calculated the mean activity at channels in the region of interest for the corresponding frequency bands: Delta: frontal, central, temporal; Theta: frontal, central; Alpha: parietal, temporal, occipital; Beta: parietal, occipital.

#### 2.7.5. Event-Related Potentials

For investigation of ERPs, epochs of 200 ms before and 800*ms* after the stimulus onset were created. We chose a more prolonged epoch duration. We decided to do so as the stimulus is continuous with multiple information to be processed. Therefore, the characteristic ERP component of interest could occur delayed. We aggregated data from all epochs to the averaged evoked response for each condition block and channel. We calculated the average activation of a specific time of interest for every condition to investigate differences in particular components. As the P300 is known to be prominent at midline electrodes, we included the channels Fz, Cz, and Pz separately in our analysis. The respective time interval considered for P300 is between 250 and 400 ms. Due to the formerly mentioned suspected occurrence delay, we also considered the time interval between 400 and 800 ms in our analysis.

#### 2.7.6. Statistical Analysis of EEG Data

Of the corresponding data, we investigated differences between the three tests conditions. We computed a Mauchly's to test if the data met the assumption of sphericity. We tested for normal distribution of the data with the Shapiro–Wilk test. If data were not normally distributed, we conducted a Friedman's test to investigate differences between conditions. If the data met the assumption of the normal distribution, we conducted a repeated-measures ANOVA with the main effect test condition. *Post-hoc* comparisons were calculated with a Wilcoxon signed-rank test.

## 3. Results

### 3.1. Subjective Data

**Figure 3:**
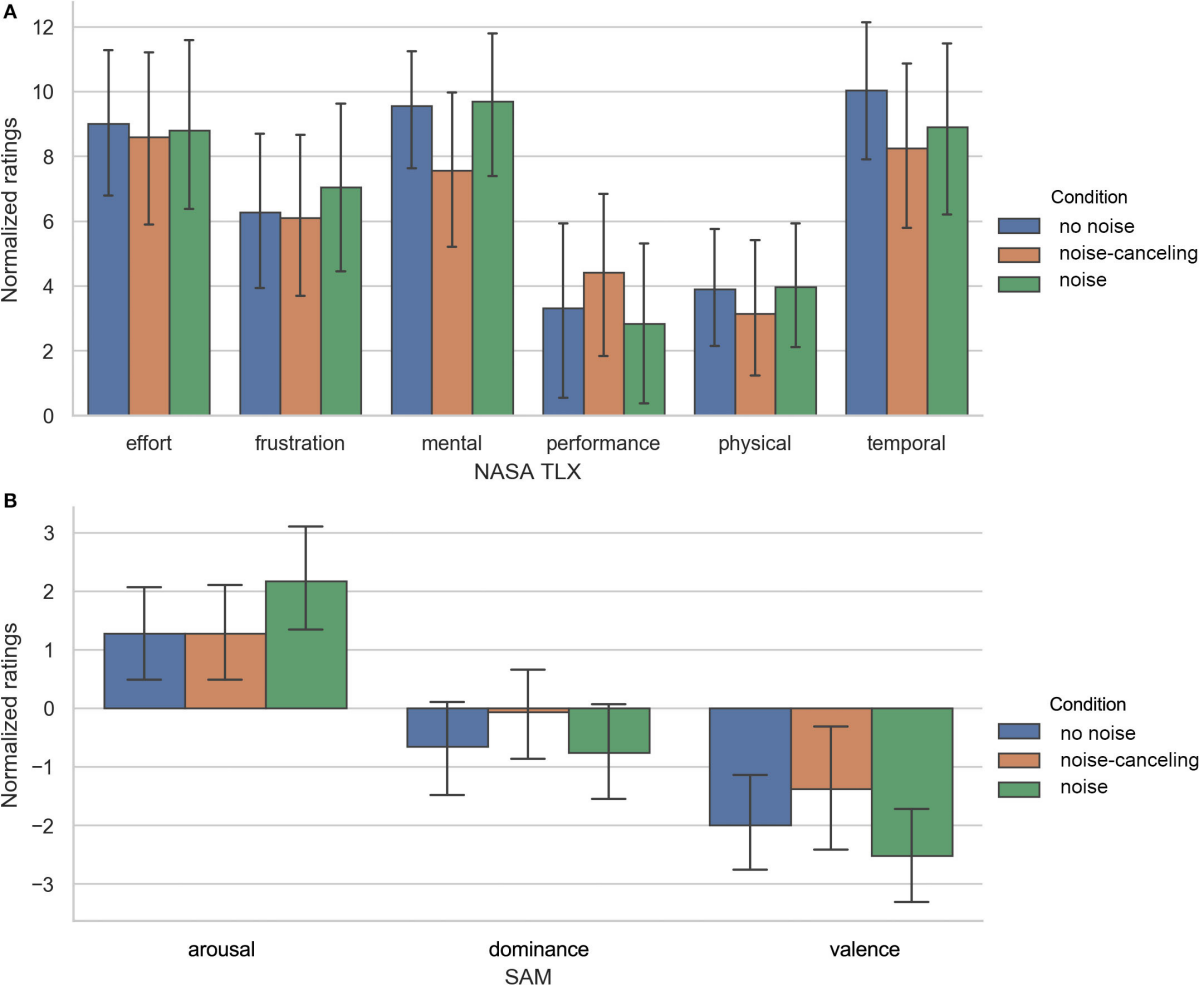
**(A)** Normalized values for NASA TLX. Ratings from *before* were subtracted from ratings *after*. Error bars show 95% confidence interval. **(B)** Normalized values for SAM. Error bars show 95% confidence interval.

The differences of ratings between before and after one main task block for NASA TLX and SAM are shown in Figure 3. Results of the repeated measures ANOVA of normalized values showed no significant main effect condition but a significant main effect “block order” for SAM item “valence” [*F*_(2, 56)_ = 4.413; *p* = 0.018; $\eta_{\text{p}}^{2}$ = 0.136]. The *post-hoc* comparison showed significantly (*p* = 0.022) lower ratings in the second compared to the first block and lower values in the last (*p* = 0.032) compared to the first block.

Although we found no other significant comparisons, on a descriptive level, the normalized values of NASA-TLX, Figure 3A show lower absolute values for negatively associated items, e.g., “effort” and “frustration” in the *noise-canceling* condition. This goes along with higher ratings in the positive-related item “performance” (NASA TLX) and lower decreases in “dominance,” “valence” (SAM) (see Figure 3B) and the rating of the subjective rating scale.

Table 1 shows M and SD of ratings for every item before and after the main task. The values suggest that participants felt more mentally loaded or rather more uncomfortable after the task than before. Out of that, we can assume that the main task seems to be demanding in every condition. This observation is supported by the normalized ratings of the items “mental,” “effort,” and “temporal” (NASA TLX), which reached the highest scores of the NASA TLX. As the comparisons between conditions are not significant, it seems as if the participants invested similar effort in all test conditions. Interestingly, the participants reported the highest temporal pressure in the *no noise* condition.

**Table 1:** Mean and standard deviation of subjective measure ratings before and after the performed task.

		**Condition**											
		**no noise**				**noise-canceling**				**noise**			
		**Before**		**After**		**Before**		**After**		**Before**		**After**	
**Questionnaire**	**Item**	**M**	**SD**	**M**	**SD**	**M**	**SD**	**M**	**SD**	**M**	**SD**	**M**	**SD**
NASA TLX	Mental	6.14	5.36	15.69	4.09	9.07	5.92	16.62	3.65	6.97	5.23	16.66	3.94
	Physical	2.66	2.91	6.55	5.68	3.93	4.63	7.07	6.37	2.79	3.03	6.76	5.65
	Temporal	6.86	6.40	16.90	2.62	9.31	6.47	17.55	2.26	7.66	6.37	16.55	3.51
	Performance	8.62	6.29	11.93	5.27	8.24	5.57	12.66	4.70	9.14	5.26	11.97	5.68
	Effort	7.31	6.46	16.31	3.49	8.14	6.36	16.72	2.45	8.41	6.62	17.21	2.97
	Frustration	8.17	6.38	14.45	4.76	8.52	5.84	14.62	3.86	8.03	5.98	15.07	3.99
SAM	Valence	5.86	1.77	3.86	2.15	5.79	1.97	4.41	1.88	6.24	1.62	3.72	1.98
	Arousal	4.24	1.92	5.52	2.03	4.48	1.90	5.76	2.05	3.90	1.76	6.07	1.81
	Dominance	4.72	1.67	4.07	1.85	4.76	1.62	4.69	1.77	4.76	1.46	4.00	1.58
SRS		2.79	1.80	5.55	1.27	3.48	1.94	5.79	1.18	3.21	1.88	5.90	1.21

### 3.2. EEG Data

Due to the individual response times for each equation and the therefore varying overall number of quotations per condition, the count of trials per condition considered for analysis varied: *no noise* condition (*M* = 253.97, *SD* = 78.17, *min* = 129, *max* = 406); *noise-canceling* condition (*M* = 267.21, *SD* = 82.82, *min* = 111, *max* = 430), and *noise* condition (*M* = 259.62, *SD* = 75.89, *min* = 121, *max* = 410).

#### 3.2.1. Frequency

From the repeated measures ANOVA results for the frequency band delta, we cannot reject the null hypothesis in favor of the alternate hypothesis [*F*_(2, 54)_ = 1.488, *p* = 0.235, $\eta_{\text{p}}^{2}$ = 0.052]. There is no significant differences between the average values of the frequency band conditions. Similar results were obtained for theta [*F*_(2, 54)_ = 0.426, *p* = 0.655, $\eta_{\text{p}}^{2}$ = 0.016], Beta [*F*_(2, 54)_ = 0.708, *p* = 0.497, $\eta_{\text{p}}^{2}$ = 0.026] and Gamma [*F*_(2, 54)_ = 1.933, *p* = 0.155, $\eta_{\text{p}}^{2}$ = 0.067].

**Figure 4:**
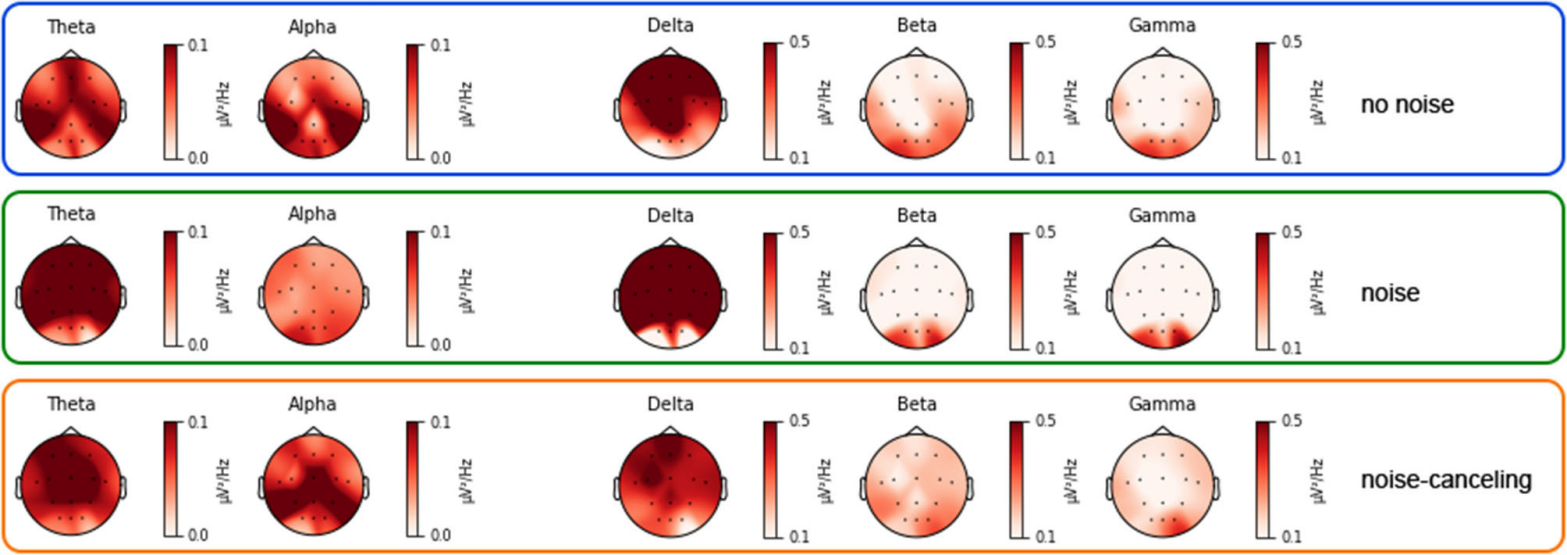
Topographies of the different frequency bands for *noise, no noise*, and *noise-canceling* condition. The red colored intensity shows the normalized power spectral density values. The scaling of the intensities was adjusted according to the frequency bands.

For alpha, the p values of Shapiro–Wilk tests were partly significant (*p* < 0.001, *p* < 0.079, *p* = 0.12) for one of the three levels of the condition-factor. As the assumption normal distribution is violated we computed a Friedman test, which revealed no significant difference between the conditions $\chi_{\left(2\right)}^{2}=3.5$, *p* = 0.174. Figure 4 shows the topography plots for every condition and every frequency band, respectively. On a descriptive level, the intensities show the hypothesized behavior of higher power in Theta and Delta, frequency band for *noise* compared to the other two conditions. Also Alpha power spectral density seems to be smallest in the *noise* condition. But these observations aren't supported by statistical significant results. The spectral power densities in Beta and Gamma frequency band show only very minimal changes in intensity between the conditions.

#### 3.2.2. Event-Related Potentials

##### 3.2.2.1. Time Interval 250–400 ms After Stimulus Onset

**Figure 5:**
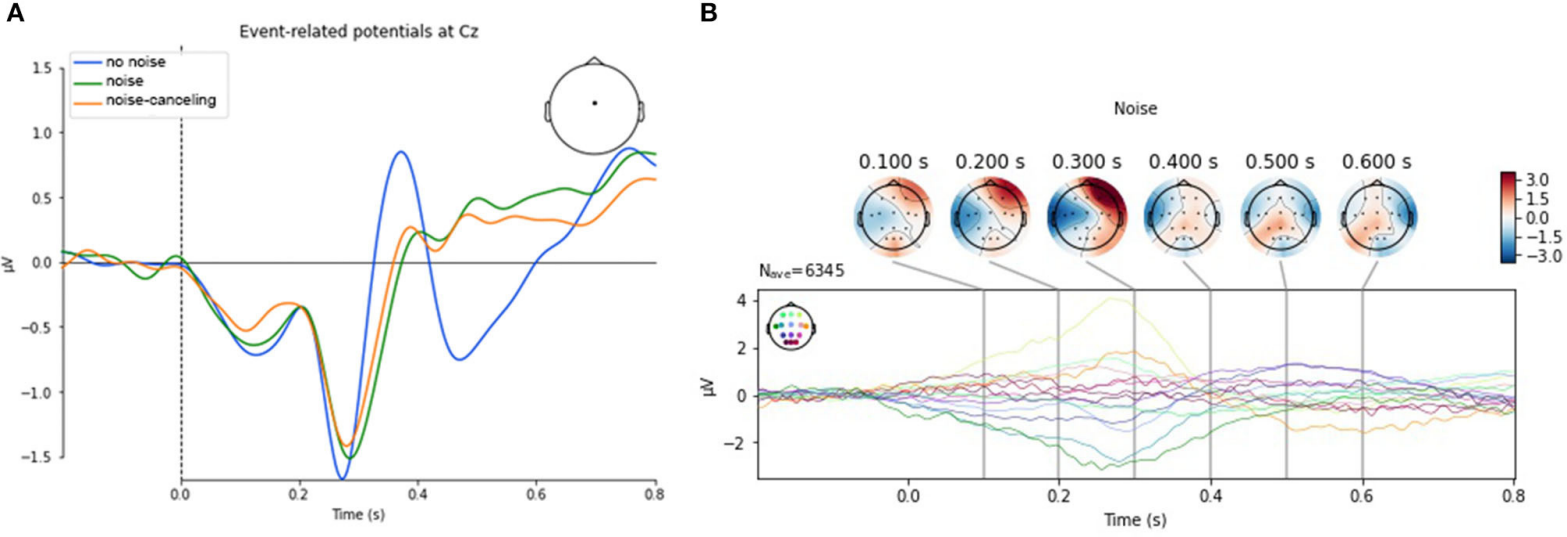
**(A)** Event-related potentials at Cz. The characteristic signal course of component of P300 the amplitude is higher in *no noise* compared to the other two conditions. **(B)** Topography plot of event-related potentials aggregated from all sensors exemplary for the *noise* condition. Sensor positions are color-coded. The activity range is given from −3 to 3μV. After 400*ms* after stimulus, the activation is increasingly prominent at the centro-parietal area of the scalp that indicates a P300 response.

From the repeated measures ANOVA results for Fz, we cannot reject the null hypothesis in favor of the alternate hypothesis [*F*_(2, 54)_ = 1.601, *p* = 0.211, $\eta_{\text{p}}^{2}$ = 0.056]. We conclude that the mean P300 activation at Fz does not significantly differ between the conditions. The measure of effect size (partial eta squared; $\eta_{\text{p}}^{2}$ = 0.056) suggests that there is a negligible effect of the conditions on the P300 activation. Mauchly's test of sphericity for Cz revealed a significant *p*-value (*p* = 0.006). Hence the data did not meet the assumption of sphericity. As the assumptions of the repeated measures ANOVA were violated, we ran a Friedman's test to investigate a main effect of the condition. The Friedman's Test showed a significant difference between the three conditions [$\chi_{\left(2\right)}^{2}=12.214$, *p* = 0.002]. A pair-wise comparison using Wilcoxon signed-rank tests between the conditions revealed significant differences between the *noise* and the *no noise* condition (*W* = 76, *p* = 0.004), with a higher amplitude for *no noise* (*Mdn* = 0.04) compared to *noise* (*Mdn* = −0.10). This difference is remarkable visible in Figure 5A. Also the difference between the *noise-canceling* (*Mdn* = −0.58) and the *no noise* (*Mdn* = 0.04) condition reached statistical significance (*W* = 82, *p* = 0.006). The difference between *noise* and *noise-canceling* is not statistically significant (*W* = 198, *p* = 0.909). We conclude that the data at Pz is non-normally distributed as the *p*-values of Shapiro–Wilk tests are partly significant (*p* < 0.001, *p* < 0.001, *p* = 0.15) for two of the three levels of the condition factor. As the assumptions of the repeated measures ANOVA were violated, we ran a Friedman's test to investigate a main effect of the condition. The Friedman's test showed no significant difference between the three conditions, $\chi_{\left(2\right)}^{2}=2$, *p* = 0.368.

##### 3.2.2.2. Time Interval 400–800 ms After Stimulus Onset

From the repeated measures ANOVA results for Fz, we cannot reject the null hypothesis in favor of the alternate hypothesis [*F*_(2, 54)_ = 1.194, *p* = 0.311, $\eta_{\text{p}}^{2}$ = 0.042]. We conclude that the mean activation at Fz does not significantly differ between the conditions.

As the assumptions of the repeated measures ANOVA is violated (evidence for a violation of the assumption of sphericity through Mauchly's test at *p* < 0.001), we ran a Friedman's test to investigate the main effect of the condition at Cz. The Friedman's test shows a significant difference between the three conditions [$\chi_{\left(2\right)}^{2}=7.358$, *p* = 0.025]. *Post-hoc* tests using a Wilkoxon signed-rank test shows that the activation in the *noise* condition (*Mdn* = 0.28) is higher than in the *no noise* condition (*Mdn* = −0.08). This differences is statistically significant (*Z* = 107, *p* = 0.029). However, the differences between *noise* and *noise-canceling* (*Mdn* = 0.17) are statistically non-significant (*Z* = 138, *p* = 0.139). Likewise, the difference in the *no noise* and the *noise-canceling* is not significant either (*Z* = 151, *p* = 0.236).

As the assumptions of the repeated measures ANOVA for Pz are violated [evidence for a violation of the assumption of sphericity through Mauchly's test (*p* < 0.001)], we ran a Friedman's test to investigate a main effect of the condition. The Friedman's test shows a non-significant difference between the three conditions [$\chi_{\left(2\right)}^{2}=1.786$, *p* = 0.409]. All results can also be found in Table 2 for a better overview. Figure 5B shows the topographies of ERPs from all sensors exemplary for the *noise* condition. Additional plots of event-related potentials at midline electrodes can be found in Figures S1–S3. Topographies of the evoked responses of all electrodes can be found in Figure S4.

**Table 2:** Statistical results of event-related potentials (ERPs) with different periods and the three midline sensors.

**Time**	**Sensor**	**Main effect**	***Post-hoc***			**Median**		
			***n - nn***	***n - nc***	***nn - nc***	**nn**	**n**	**nc**
250–400 ms	Fz	*F*_(2, 54)_ = 1.601, *p* = 0.211, $\eta_{\text{p}}^{2}$ = 0.056						
	Cz	$\chi_{\left(2\right)}^{2}=12.214$, ***p*** **= 0.002**	***W*** **= 76**, ***p*** **= 0.004**	*W* = 198, *p* = 0.909	***W*** **= 82**, ***p*** **= 0.006**	0.04	−0.10	−0.58
	Pz	$\chi_{\left(2\right)}^{2}=2$, *p* = 0.368						
400–800 ms	Fz	*F*_(2, 54)_ = 1.194, *p* = 0.311, $\eta_{\text{p}}^{2}$ = 0.042						
	Cz	$\chi_{\left(2\right)}^{2}=7.358$, ***p* = 0.025**	***Z*** **= 107**, ***p*** **= 0.029**	*Z* = 138, *p* = 0.139	*Z* = 151, *p* = 0.236	−0.08	0.28	0.17
	Pz	$\chi_{\left(2\right)}^{2}=1.786$, *p* = 0.409						

*In case of significant results, values are marked in bold. For significant post-hoc comparisons, median values of every condition are given*.

## 4. Discussion

### 4.1. Subjective Assessment

The significant main effect of block order for the item “valence” (SAM) indicates that the participants felt less positive and therefore were potentially more uncomfortable in the later stages of the experiment. According to descriptive values of items “temporal” and “mental,” the participants seemed to perceive high mental demand and time pressure. Based on descriptive values (higher ratings in positive items and lower ratings in negative items during *noise-canceling*, see section 3.1), we conclude that the subjectively experienced increase of, e.g., mental demand or stress is lower in the *noise-canceling* condition. However, this assumption has to be further substantiated in future studies. Surprisingly, we could not find a similar observation in the *no noise* condition. A possible explanation could be an unexpected psychological influence reported verbally by few participants: in a quiet environment, they felt more uncomfortable than in a noisy environment. Potentially, the feeling of being observed increased the pressure to perform well due to no excuse for making mistakes. The presence of an experimenter is necessary to guide and maintain the experiment procedure especially when working with physiological measures. More importantly, we ensure to minimize the feeling of being observed by placing the experimenters to not look at the participants' screen and by emphasizing that the experimenter did not observed them directly throughout the experiment. This should mimic a general office situation with colleagues nearby but without direct monitoring.

The fact that the differences between the silent (*no noise*) and the noisy (*noise* and *noise-canceling*) conditions indicated in the data did not reach significance could be justified in the relatively short test block duration. A prolongation of each test condition duration could increase the already visible (but not significant) differences between the conditions. Whereby, this adjustment could also cause other influence factors like fatigue, which are hard to control.

### 4.2. Brain Activity

The analysis of the spectral power of frequency bands delivered no statistical evidence for differences between conditions. The differences in activity between the conditions that are indicated in Figure 4 reach no significant value.

The analysis of ERPs, however, revealed interesting results. We suggested a decrease in amplitude of the P300 peak amplitude with increasing noise levels. Our results support the assumption that the P300 amplitude is highest in the *no noise* condition at electrode Cz compared to the other two. The difference between the *no noise* and *noise* condition, as well as between *no noise* and *noise-canceling* was found to be statistically significant in the time 250– 400 ms after stimulus onset. After the peak in amplitude in the *no noise* condition, the signal drops rapidly with a negative peak around 500*ms* after stimulus onset. In the other noisy conditions, the activation stays positive and even slightly increases. The difference reaches significance between the *no noise* and the *noise* condition. The formerly mentioned observation that the P300's latency remains larger with a higher mental workload could explain this behavior, which would support our hypothesis of higher mental demand in the *noise* condition. The topography of the ERP, as shown in Figure 5B, supports this assumption.

A significant discrimination between *noise-canceling* and *noise* could not be found in the ERPs. This lack of differentiation could have several reasons. The present experiment presented the target stimuli (arithmetic equations) directly on both ears via headphones. The distracting stimuli was present as ambient sound but also detectable for both ears similar. Additionally, the target and distraction stimuli were complex as they consisted of speech and environmental noise, which varies in frequency and inter-stimulus intervals. This frequency and time-varying presentation of stimuli could affect the brain activity-related components in several ways. For example, a jittering in stimulus presentation is known to reduce the peak amplitude of ERPs. Furthermore, the resulting timing effect of ERPs can shift and be later compared to non-jittered stimulus presentations due to jittering in timing. Due to the aforementioned multiple ways how the ERP components can be influenced, an interpretation of a substantial influence is difficult.

Further investigation should focus on more significant discrimination between the two noisy test situations. Our approach resulted in overall high demand (according to ratings in NASA item “mental”) in all conditions, making it hard to discriminate between the different experimental manipulations. One reasonable modification would be choosing a visual first task, for example, reading, and adding a second task like detecting specific auditory events (“auditory oddball”; for more details, see Duncan et al., 2009). This would address two modalities and reduce the demand in one channel while keeping the overall demand high. The second task would demand additional attentional resources. This testing paradigm would still be comparable to a real-life working situation (e.g., mobile working on a business trip) in which a person is focused on the work but must not miss important announcements. Additionally, this approach has the advantage of clear differentiation in brain characteristics, becoming more straightforward than the present task.

### 4.3. Limitations

The study has some already mentioned limitations regarding the setup and the stimuli, which should be addressed in further studies. Regarding the processing of the EEG data, the main focus was on the sensor-based analytic. It would be possible also to consider doing the performed data analysis on source signals obtained by a source reconstruction. Of course, that approach is limited due to the number of used electrodes in the current setup. It would be advisable to increase the number of electrodes in total or focus on specific cortical areas known to show the observed effects.

### 4.4. Conclusion

The current study aimed to investigate differences in the mental load of participants in varying environmental noise situations. Moreover, it was of interest if noise-canceling headphones help to reduce mental load while focusing on a task. We suggested finding indications that the *noise* condition results in a higher mental load than the other two conditions. The noise-canceling technology was suggested to improve the user's situation in terms of mental load and stress. Additionally, we assumed that in the *no noise* condition, the participants felt less loaded as in the *noise-canceling* condition. We found evidence in subjective data that valence decreases from the beginning to the end of the experiment.

The ERPs of electrical brain activity resulted in significant differentiation between the *no noise* and the other two test conditions. The mentioned adjustment of the setup and the analysis could lead to a stronger delimitation of the two noisy situations. The findings of the current work provide a foundation for the investigation of noise-cancelation and its potential improvement of the working situation in noisy surroundings.

## Data Availability Statement

The datasets presented in this article are not readily available because the raw data access is restricted by a disclosure statement with the funder. Requests to access the datasets should be directed to kerstin.pieper@tu-berlin.de.

## Ethics Statement

The studies involving human participants were reviewed and approved by Ethics Committee of Faculty IV—Electrical Engineering and Computer Science at the Technical University of Berlin. The patients/participants provided their written informed consent to participate in this study.

## Author Contributions

KP, SM, EP, MV, and J-NV-A contributed to the design of the study. KP, RS, and PP implemented the stimuli, carried out data collection, and conducted data analyses. KP wrote the main manuscript text. All authors reviewed and contributed to the final manuscript.

## Funding

This research was funded by Tampere Wireless Headset Audio Lab, Finland Research Center, Huawei Technologies Oy (Finland) Co., Ltd., Tampere, Finland.

## Conflict of Interest

EP and MV were employed by the company Tampere Wireless Headset Audio Lab, Finland Research Center, Huawei Technologies Oy (Finland) Co., Ltd., Tampere, Finland. PP was employed by Pupil Labs GmbH, Berlin, Germany. The remaining authors declare that this study received funding from Tampere Wireless Headset Audio Lab, Finland Research Center, Huawei Technologies Oy (Finland) Co., Ltd., Tampere, Finland. The funder was not involved in collection, analysis, and interpretation of data.

## Publisher's Note

All claims expressed in this article are solely those of the authors and do not necessarily represent those of their affiliated organizations, or those of the publisher, the editors and the reviewers. Any product that may be evaluated in this article, or claim that may be made by its manufacturer, is not guaranteed or endorsed by the publisher.

## Footnotes

^1^ https://github.com/labstreaminglayer/App-g.Tec/releases/tag/gusbamp

^2^ https://github.com/chkothe/pylsl

^3^ https://www.itu.int/rec/T-REC-P.910-200804-I/en

^4^ https://www.itu.int/rec/T-REC-P.911-199909-I!Cor1/en
