# Supplementary material for: Working With Environmental Noise and Noise-Cancelation: A Workload Assessment With EEG and Subjective Measures
Source: Front Neurosci. 2021 Nov 1;15:771533. doi: 10.3389/fnins.2021.771533 (PMC8591241; doi:10.3389/fnins.2021.771533)
Supplement: Supplementary file 1 [file Data_Sheet_1.pdf]

frontiers

Supplementary Material

1 SUPPLEMENTARY FIGURES

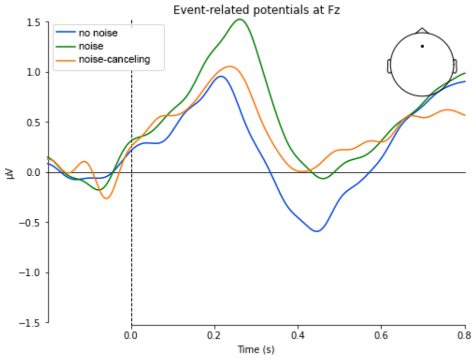

Figure S1. Event-related potentials at Fz. The signal amplitude seems to be higher in *noise* compared to the other two conditions but this difference is not significant.

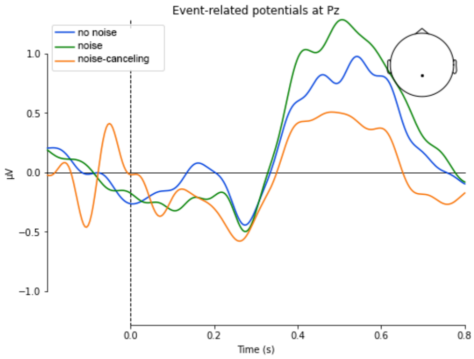

Figure S2. Event-related potentials at Pz. A positive fluctuation gets visible later compared to Fz and Cz.

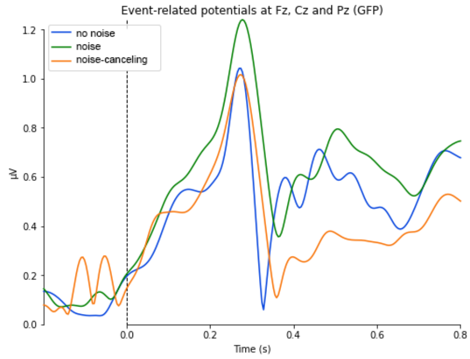

Figure S3. Event-related potentials aggregated from the three midline electrodes (Fz, Cz, and Pz) by Global Field Power (GFP).

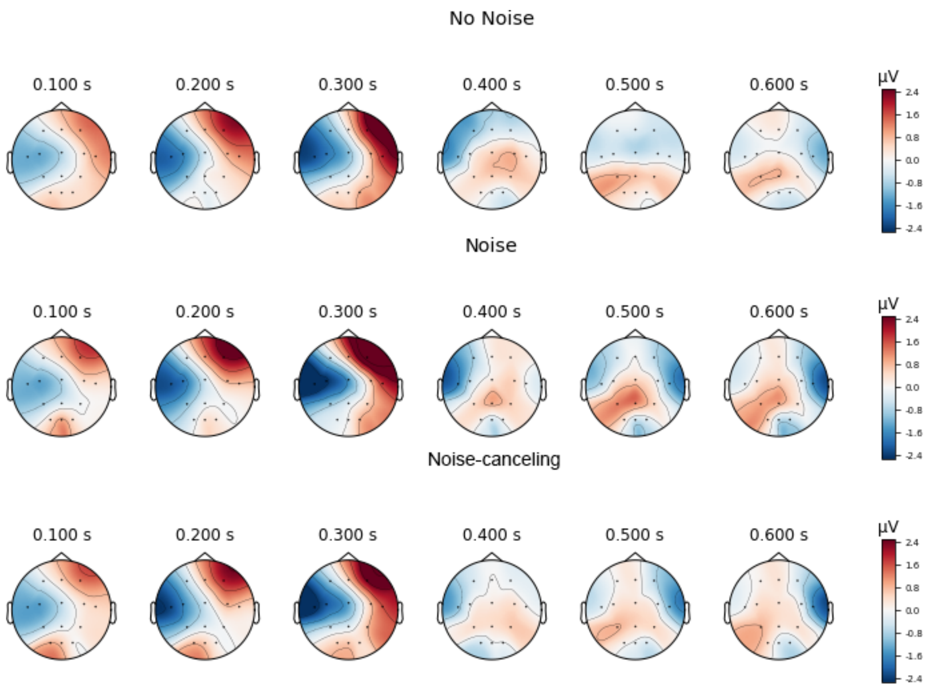

Figure S4. Topographies of the evoked responses of all electrodes in the respective condition.
